# Occurrence data uncover patterns of allopatric divergence and interspecies interactions in the evolutionary history of *Sceloporus* lizards

**DOI:** 10.1002/ece3.7237

**Published:** 2021-02-08

**Authors:** Julio A. Rivera, Heather N. Rich, A. Michelle Lawing, Michael S. Rosenberg, Emília P. Martins

**Affiliations:** ^1^ School of Life Sciences Arizona State University Tempe AZ USA; ^2^ Department of Ecosystem and Science Management Texas A&M University College Station TX USA; ^3^ Center for the Study of Biological Complexity Virginia Commonwealth University Richmond VA USA

**Keywords:** bioregions, infomaps, phenotypic evolution, sceloporus, sympatry, trait divergence

## Abstract

As shown from several long‐term and time‐intensive studies, closely related, sympatric species can impose strong selection on one another, leading to dramatic examples of phenotypic evolution. Here, we use occurrence data to identify clusters of sympatric *Sceloporus* lizard species and to test whether *Sceloporus* species tend to coexist with other species that differ in body size, as we would expect when there is competition between sympatric congeners. We found that *Sceloporus* species can be grouped into 16 unique bioregions. Bioregions that are located at higher latitudes tend to be larger and have fewer species, following Rapoport's rule and the latitudinal diversity gradient. Species richness was positively correlated with the number of biomes and elevation heterogeneity of each bioregion. Additionally, most bioregions show signs of phylogenetic underdispersion, meaning closely related species tend to occur in close geographic proximity. Finally, we found that although *Sceloporus* species that are similar in body size tend to cluster geographically, small‐bodied *Sceloporus* species are more often in sympatry with larger‐bodied *Sceloporus* species than expected by chance alone, whereas large‐bodied species cluster with each other geographically and phylogenetically. These results suggest that community composition in extant *Sceloporus* species is the result of allopatric evolution, as closely related species move into different biomes, and interspecies interactions, with sympatry between species of different body sizes. Our phyloinformatic approach offers unique and detailed insights into how a clade composed of ecologically and morphologically disparate species are distributed over large geographic space and evolutionary time.

## Graphical abstract

Here, we use open source databases to understand the evolutionary impact of sympatric species on phenotypes. We found that species are not evenly distributed across the Americas and that small‐bodied lizard species are geographically overdispersed and are often found in sympatry with species of a different size class. Conversely, large‐bodied species cluster in geographic space and are often in sympatry with other large species

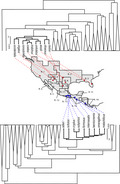

## 1 INTRODUCTION

The current availability of geographically informed, organismal data offers opportunities for new insights into evolutionary processes such as trait divergence and sympatric diversification that have been traditionally studied through time‐intensive fieldwork. Often, selective forces lead to phenotypes that allow species to occupy distinct ecological niches while living in sympatry with other closely related species (Lack, 1947; López Juri et al., 2015; Pacala & Roughgarden, 1982). This is particularly apparent in adaptive radiations in which species evolve specialized morphologies that allow them to exploit distinct microhabitats. For example, the Caribbean anoles have evolved disparate limb morphologies that allow them to occupy different ecological niches, thereby reducing competition (Losos, 1992; Losos et al., 1998). Similar examples of adaptive radiation and convergent evolution (ecomorphs) have been found in other island species (Darwin's finches: Grant & Grant, 2006, Hawaiian silverswords: Blonder et al., 2016, Hawaiian spiders: Gillespie, 2004) and on larger scales, for example, in Europe and North America fishes (Lamouroux et al., 2002). Coexistence with closely related species and niche‐partitioning are clearly also important drivers for mainland communities (Kartzinel et al., 2015; Mujic et al., 2016), but their effects can be difficult to detect, for example, as in the composition of plants in the Amazon rain forest (Kraft & Ackerly, 2010) and the assemblage of bat species on a latitudinal gradient across the Americas (Stevens et al., 2003). In part, these mainland community patterns are obscured by power limitations of the existing methods (Kraft & Ackerly, 2010) and the relatively small sample sizes imposed by the time‐intensive nature of collecting the necessary data. Here, we use the power of massive data available in online databases and recent advances in data manipulation and statistical modeling to study patterns of sympatry and body size across a diverse genus of lizards.

Gause's competitive exclusion principle dictates that two species occupying the same niche should not be able to coexist (Gause, 1934; Mayfield & Levine, 2010). However, it has been long known that closely related taxa occur together more often than expected by chance alone (Elton, 1946; Moreau, 1948; Williams, 1947) and may also expand the range of physical conditions in which other species can live (e.g., by providing refuges: Bulleri et al., 2016) or shape phenotypes through character displacement (Dayan & Simberloff, 2005; Losos, 2000; Moritz et al., 2018; Schluter & McPhail, 1993). For example, the plethodontid salamanders, *Plethodon hoffmani* and *P. cinereus,* are identical when living in allopatry (Adams & Rohlf, 2000). However, when the two species are in sympatry, they exhibit differences in jaw morphology that allow them to partition their prey resources so that *P. hoffmani* eats larger, faster prey while *P. cinereus* eats smaller, slower prey (Adams, 2004). Sympatric European lacertid lizards similarly exhibit small changes in head morphology that allow the partitioning of food resources by species, sexes, and age category (Herrel et al., 2001). Other studies have emphasized evolution via selective migration (Gillespie, 2004; Li et al., 2015), phenotypic convergence in older lineages (Tobias et al., 2014), and interactions with phylogenetically unrelated competitors (Wilcox et al., 2018). Theoretical discussions of these forces (e.g., Edwards et al., 2018; Mittelbach and Schemske 2015) suggest that there is still much to learn about the selective impact of phenotypically similar sympatric taxa.

Body size is a convenient and effective trait for analyzing the relationship between abiotic factors, like climate, and morphology (Bergmann, 1847), and often serves as a starting point to understand trait divergence in sympatric species (Echeverría‐Londoño et al., 2018; Thomas et al., 2009). The relationship between body size and sympatry has been studied in many groups of amphibians and reptiles (Dunham et al., 1978; Kozak et al., 2009; Moen & Wiens, 2009; Okuzaki et al., 2010; Soule, 1966). For example, in the Australian *Gehyra* lizards, differences in body size are greater in sympatric lineages than in allopatric lineages (Moritz et al., 2018) suggesting that body size divergence is a key element of the process by which species coexist.

The genus *Sceloporus* serves as an ideal system to study the forces involved in the evolution of species in sympatry and how body size is distributed over geographic space and evolutionary time. *Sceloporus* lizards, also known as spiny or fence lizards, have been well studied in terms of behavior (Carpenter et al., 1977; Hews et al., 2013), physiology (Angilletta et al., 2004; Beal et al., 2014), and phylogenetic relationships (Leaché et al., 2016; Sites et al., 1992; Wiens et al., 2010). Moreover, the genus *Sceloporus* has a large geographic distribution that encompasses much of North America and Central America (Sites et al., 1992), is speciose with nearly 100 species (Leaché et al., 2016), and possesses a tremendous amount of ecological diversity inhabiting lowland deserts, high‐elevation alpine forests, and grasslands (IUCN, 2018). *Sceloporus* species also can be found in sympatry with other congeners at varying degrees, but this has only been studied at a small, local scale (Grummer et al., 2015; Serrano‐Cardozo et al., 2008). Although the genus *Sceloporus* does not possess a tremendous amount of morphological disparity, it offers an intriguing opportunity for discovery analyses using publicly available data over a large geographic area.

Here, we use information from large, open‐source databases to study the biogeographic and phenotypic patterns of congeneric sympatry among *Sceloporus* species. First, we use spatial distribution data and phylogenies to ask how species are spatially grouped, and how sympatry relates to phylogenetic divergence. Specifically, do closely related species cluster geographically or are they evenly spread across the landscape? In particular, we apply a bioregions approach (Vilhena & Antonelli, 2015). Bioregions are a powerful way to describe species distributions and can be used in evolutionary biology to infer historical processes that led to current day distributional patterns (Matzke, 2014; Ree & Smith, 2008). We also test whether characteristics of each geographic area correlate with species richness to determine if particular factors are contributing to the biogeographic patterns of the clade. Then, we infer how body size is distributed over different spatial regions and has evolved through time. Finally, we combine occurrence data, phylogenies, and body size to ask whether species of different body sizes co‐occur more often than by chance to understand how phenotypic diversity varies across space.

## 2 MATERIAL AND METHODS

### 2.1 The biogeography of congeneric sympatry

We obtained point‐occurrence data for 69 species of *Sceloporus* lizards from the Global Biodiversity Information Facility (Tgbif, 2018), using the “rgbif” v0.9.9 package (Chamberlain,) in R (R Core Team, 2017). The most recent phylogenetic analysis of the genus by Leaché, Banbury (Leaché et al., 2016) identifies 97 *Sceloporus* species and sampled 86 species to construct the phylogeny. Our study includes 69 of these species that are also represented in GBIF. The remaining species are known from very few records. The 13 species that are identified by the Leaché, Banbury (Leaché et al., 2016) phylogeny but not in our current study are roughly evenly distributed geographically, phylogenetically, and by size, such that excluding them from our analyses is unlikely to bias our results. For each species in our study, we removed all points that were likely errors, clearly falling outside of the geographic ranges provided by International Union for Conservation of Nature (IUCN, 2018). Our final data set consisted of 133,665 occurrence points for the 69 species (Table 1).

**Table 1:** Number of occurrence points used to characterize the range of each species, the mean Snout‐to‐Vent Length (SVL, with number of individuals used to obtain that estimate in parentheses), and the published reference from which we obtained body size data. Also included are size data for the outgroup used in the ancestral reconstruction analysis

Species	*N* occurrence points	Average SVL (mm) (*N*)	Source
*Sceloporus adleri*	426	65.3 (14)	Fitch (Fitch, 1978)
*Sceloporus aeneus*	2035	53.0 (148)	Jiménez‐Arcos, Sanabria‐Urbán (Jiménez‐Arcos et al., 2017)
*Sceloporus arenicolus*	141	54.5 (507)	Jiménez‐Arcos, Sanabria‐Urbán (Jiménez‐Arcos et al., 2017)
*Sceloporus bicanthalis*	326	43.6 (56)	Jiménez‐Arcos, Sanabria‐Urbán (Jiménez‐Arcos et al., 2017)
*Sceloporus bulleri*	140	100.7 (10)	Fitch (Fitch, 1978)
*Sceloporus carinatus*	397	52.9 (2)	Rivera *et al*., unpublished
*Sceloporus cautus*	341	67.9 (11)	Smith (Smith, 1939)
*Sceloporus chrysostictus*	2089	54.0 (163)	Fitch (Fitch, 1978)
*Sceloporus clarkii*	653	103.0 (56)	Jiménez‐Arcos, Sanabria‐Urbán (Jiménez‐Arcos et al., 2017)
*Sceloporus consobrinus*	1888	60.3 (45)	Jiménez‐Arcos, Sanabria‐Urbán (Jiménez‐Arcos et al., 2017)
*Sceloporus couchii*	827	58.0 (32)	Jiménez‐Arcos, Sanabria‐Urbán (Jiménez‐Arcos et al., 2017)
*Sceloporus cowlesi*	1,043	62.5 (2)	Rivera *et al*., unpublished
*Sceloporus cryptus*	58	61.6 (6)	Jiménez‐Arcos, Sanabria‐Urbán (Jiménez‐Arcos et al., 2017)
*Sceloporus cyanogenys*	1,066	96.0 (8)	Jiménez‐Arcos, Sanabria‐Urbán (Jiménez‐Arcos et al., 2017)
*Sceloporus dugesii*	393	65.9 (7)	Jiménez‐Arcos, Sanabria‐Urbán (Jiménez‐Arcos et al., 2017)
*Sceloporus edwardtaylori*	143	107.0 (?)	Jiménez‐Arcos, Sanabria‐Urbán (Jiménez‐Arcos et al., 2017)
*Sceloporus formosus*	1,106	68.0 (111)	Jiménez‐Arcos, Sanabria‐Urbán (Jiménez‐Arcos et al., 2017)
*Sceloporus gadoviae*	606	64.9 (6)	Jiménez‐Arcos, Sanabria‐Urbán (Jiménez‐Arcos et al., 2017)
*Sceloporus graciosus*	10,982	55.2 (182)	Jiménez‐Arcos, Sanabria‐Urbán (Jiménez‐Arcos et al., 2017)
*Sceloporus grammicus*	15,050	60.1 (412)	Jiménez‐Arcos, Sanabria‐Urbán (Jiménez‐Arcos et al., 2017)
*Sceloporus grandaevus*	835	72.1 (5)	Jiménez‐Arcos, Sanabria‐Urbán (Jiménez‐Arcos et al., 2017)
			
*Sceloporus heterolepis*	55	59.7 (37)	Smith (Smith, 1939)
*Sceloporus horridus*	2,825	85.5 (82)	Jiménez‐Arcos, Sanabria‐Urbán (Jiménez‐Arcos et al., 2017)
*Sceloporus hunsakeri*	641	73.9 (20)	Jiménez‐Arcos, Sanabria‐Urbán (Jiménez‐Arcos et al., 2017)
*Sceloporus insignis*	63	89.5 (10)	Fitch (Fitch, 1978)
*Sceloporus internasalis*	174	80.1 (2)	Rivera *et al*., unpublished
*Sceloporus jalapae*	582	49.3 (17)	Jiménez‐Arcos, Sanabria‐Urbán (Jiménez‐Arcos et al., 2017)
*Sceloporus jarrovii*	3,825	69.7 (668)	Jiménez‐Arcos, Sanabria‐Urbán (Jiménez‐Arcos et al., 2017)
*Sceloporus licki*	432	71.4 (24)	Jiménez‐Arcos, Sanabria‐Urbán (Jiménez‐Arcos et al., 2017)
*Sceloporus maculosus*	133	48.4 (7)	Smith (Smith, 1939)
*Sceloporus magister*	4,807	111.5 (53)	Jiménez‐Arcos, Sanabria‐Urbán (Jiménez‐Arcos et al., 2017)
*Sceloporus malachiticus*	667	79.1 (146)	Fitch (Fitch, 1978)
*Sceloporus megalepidurus*	593	47.3 (76)	Jiménez‐Arcos, Sanabria‐Urbán (Jiménez‐Arcos et al., 2017)
*Sceloporus melanorhinus*	580	84.6 (32)	Jiménez‐Arcos, Sanabria‐Urbán (Jiménez‐Arcos et al., 2017)
*Sceloporus merriami*	3,071	52.2 (355)	Jiménez‐Arcos, Sanabria‐Urbán (Jiménez‐Arcos et al., 2017)
*Sceloporus microlepidotus*	2,514	‐	NA
*Sceloporus minor*	540	70.3 (169)	Jiménez‐Arcos, Sanabria‐Urbán (Jiménez‐Arcos et al., 2017)
*Sceloporus mucronatus*	2085	87.0 (146)	Jiménez‐Arcos, Sanabria‐Urbán (Jiménez‐Arcos et al., 2017)
*Sceloporus nelsoni*	1,146	60.16 (26)	Jiménez‐Arcos, Sanabria‐Urbán (Jiménez‐Arcos et al., 2017)
*Sceloporus occidentalis*	30,367	68.4 (46)	Jiménez‐Arcos, Sanabria‐Urbán (Jiménez‐Arcos et al., 2017)
*Sceloporus ochoterenae*	348	48.2 (143)	Jiménez‐Arcos, Sanabria‐Urbán (Jiménez‐Arcos et al., 2017)
*Sceloporus olivaceus*	2,991	82.9 (34)	Jiménez‐Arcos, Sanabria‐Urbán (Jiménez‐Arcos et al., 2017)
*Sceloporus orcutti*	2,313	102 (17)	Fitch (Fitch, 1978)
*Sceloporus ornatus*	244	‐	NA
*Sceloporus palaciosi*	225	‐	NA
*Sceloporus parvus*	782	50.0 (?)	Jiménez‐Arcos, Sanabria‐Urbán (Jiménez‐Arcos et al., 2017)
*Sceloporus pictus*	110	48.9 (8)	Fitch (Fitch, 1978)
*Sceloporus poinsettii*	3,847	96.8 (79)	Jiménez‐Arcos, Sanabria‐Urbán (Jiménez‐Arcos et al., 2017)
			
*Sceloporus pyrocephalus*	462	62.0 (84)	Jiménez‐Arcos, Sanabria‐Urbán (Jiménez‐Arcos et al., 2017)
*Sceloporus scalaris*	811	45.5 (45)	Jiménez‐Arcos, Sanabria‐Urbán (Jiménez‐Arcos et al., 2017)
*Sceloporus scitulus*	328	70.2 (2)	Rivera *et al*., unpublished
*Sceloporus serrifer*	1,153	76.9 (7)	Smith (Smith, 1939)
*Sceloporus siniferus*	3,222	52.5 (235)	Jiménez‐Arcos, Sanabria‐Urbán (Jiménez‐Arcos et al., 2017)
*Sceloporus slevini*	483	47.5 (2)	Smith (Smith, 1939)
*Sceloporus smaragdinus*	539	67.2 (14)	Fitch (Fitch, 1978)
*Sceloporus smithi*	26	82.4 (57)	Smith (Smith, 1939)
*Sceloporus spinosus*	1960	92.7 (164)	Jiménez‐Arcos, Sanabria‐Urbán (Jiménez‐Arcos et al., 2017)
*Sceloporus squamosus*	733	51.6 (2)	Rivera *et al*., unpublished
*Sceloporus subpictus*	29	63.5 (1)	Jiménez‐Arcos, Sanabria‐Urbán (Jiménez‐Arcos et al., 2017)
*Sceloporus taeniocnemis*	899	77.2 (2)	Rivera *et al*., unpublished
*Sceloporus teapensis*	458	55.9 (24)	Fitch (Fitch, 1978)
*Sceloporus torquatus*	3,318	101.5 (37)	Jiménez‐Arcos, Sanabria‐Urbán (Jiménez‐Arcos et al., 2017)
*Sceloporus tristichus*	405	58.6 (24)	Fitch (Fitch, 1978)
*Sceloporus undulatus*	178	55.8 (177)	Jiménez‐Arcos, Sanabria‐Urbán (Jiménez‐Arcos et al., 2017)
*Sceloporus vandenburgianus*	663	60.2 (34)	Fitch (Fitch, 1978)
*Sceloporus variabilis*	9,255	62.0 (457)	Jiménez‐Arcos, Sanabria‐Urbán (Jiménez‐Arcos et al., 2017)
*Sceloporus virgatus*	1,299	50.4 (22)	Jiménez‐Arcos, Sanabria‐Urbán (Jiménez‐Arcos et al., 2017)
*Sceloporus woodi*	287	51.9 (78)	Jiménez‐Arcos, Sanabria‐Urbán (Jiménez‐Arcos et al., 2017)
*Sceloporus zosteromus*	1,262	103.7 (1)	Rivera *et al*., unpublished
*Petrosaurus thalassiunus*	–	110.0 (?)	Goldberg and Beaman (Goldberg & Beaman, 2004)
*Phrynosoma solare*	–	115.0 (?)	Brennan and Holycross (Brennan & Holycross, 2006)
*Urosaurus ornatus*	–	59.0 (?)	Brennan and Holycross (Brennan & Holycross, 2006)
*Uta stansburiana*	–	50.0 (?)	Stebbins (Stebbins, 1985)

We used the Infomap Bioregions application to determine how species are spatially grouped (Edler et al., 2017). We used this approach because the program uses a novel adaptive resolution method that is less sensitive to biases from incomplete species distribution data (Edler et al., 2017). Infomap Bioregions is a network approach (Vilhena & Antonelli, 2015) that uses adaptive spatial resolution to cluster point‐occurrence data from multiple species into geographic grid cells. The software creates a bipartite network of species and grid cells, connecting each species to geographic cells in which it occurs. The program then aggregates geographic cells for which there are occurrence points into larger geographic regions, creating larger regions in areas with similar species composition. The result is a detailed map with designated bioregions that are composed of unique species assemblages.

The Infomap Bioregions application has several input parameters including maximum and minimum cell size and maximum and minimum number of occurrence points per cell. We tested various combinations of parameters and found that a minimum cell size below 2° produced patchy, discontinuous bioregions. Setting the maximum cell size above 4° led to large, uninformative regions that included areas where we know that species do not co‐occur. As a result, we set the maximum cell size to 4° and the minimum cell size to 2°. Changing the maximum and minimum cell capacities (number of incident points/cell) had little impact on our findings except in slightly changing the shapes of a few bioregions. Therefore, we used the default minimum and maximum cell capacities of 100 and 10, respectively. We also provided the Leaché, Banbury (Leaché et al., 2016) phylogenetic tree as an input parameter for the analysis. Once the software produced the bioregions, we summarized the total number of biomes as described by Olson, Dinerstein (Olson et al., 2001) within each bioregion. Lastly, we used a linear model to explore the relationship between biome heterogeneity and species richness across bioregions.

We then used “phytools” v0.6–99 (Revell, 2012) in R (R Core Team, 2017) to analyze the relationship between phylogenetic relatedness and geographic distributions. First, we plotted a phylogeny of *Sceloporus* lizards (Leaché et al., 2016) onto a map of North America. Then, we linked each taxon to the map at the mean latitude and longitude for all occurrence records for that species. We omitted species that were not included in the Leaché, Banbury (Leaché et al., 2016) phylogeny. Additionally, we used the package “picante” (Kembel et al., 2010) to calculate the mean pairwise distance (MPD) between species assemblages to test whether bioregions show a pattern of phylogenetic overdispersion or underdispersion. We used the standardized effect size of the MPD and p‐value quantiles to evaluate the results.

Finally, we estimated Spearman's correlation coefficient between species richness (the number of species in a bioregion) and geographic Area (km^2^), the latitude at the region centroid (northing in km), and elevation heterogeneity in meters using a horizontal grid spacing of 30 arc seconds (the standard deviation of elevation as infered from the digital elevation model: GTOPO 30; United States Geological Survey, 2012) in R (R Core Team, 2017).

### 2.2 Body size evolution

We gathered Snout‐to‐Vent Length (SVL) measurements for adult males from each of the *Sceloporus* and outgroup species from the literature (Table 1) to test how body sizes have evolved through evolutionary time and are distributed across the phylogeny. First, we generated a histogram with the mean SVLs of each *Sceloporus* species and used the natural breaks in the data to visually generate three body size categories: large, medium, and small. We then used “phytools” v0.6–99 (Revell, 2012) in R (R Core Team, 2017) to reconstruct ancestral body sizes of the continuous SVL values using three models of evolution: Brownian motion (BM), Ornstein‐Uhlenbeck (OU), and Early‐burst (EB). We used log‐likelihoods to compare the model fit and checked for convergence. We then estimated the degree of phylogenetic signal (Pagel's λ) for the SVL size data to determine the degree to which phylogenetic history constrains body size evolution. We performed this analysis using phylogenies from both Leaché, Banbury (Leaché et al., 2016) and Wiens, Kuczynski (Wiens et al., 2010), but since our results were similar both in terms of phylogenetic signal and ancestral state reconstruction, we present only the results using the Leaché, Banbury (Leaché et al., 2016) phylogeny here.

### 2.3 Sympatry and body size evolution

For each species, we estimated the degree to which the focal taxon occurs with species of the same or different body size. To do this, we used a custom‐built Python program (Python Core Team, 2018) to assemble lists of all pairwise distances between the 133,665 geographic occurrence points. Then, for each *Sceloporus* species, we compiled a list of sympatric congeners, characterizing each species as being sympatric with a second species if at least 10 occurrence points for the target species were 5 km or closer to an occurrence point for the second species. We then added in the body size data, categorizing each species as small, medium, or large, and counting the total number of *Sceloporus* species from each body size category occurring in sympatry with each target species. Finally, we calculated a measure of “Divergent Sympatry” for each species: a chi‐squared value comparing the number of small, medium, and large sympatric congeners with the expected number of each size class given the distribution of sizes across the genus. We multiplied this measure by −1 if the difference between observed and expected counts was due to the target species being found more commonly with similarly sized congeners for clarity in the visualization of these results. We considered the distribution of body sizes at the level of bioregions, asking whether the observed number of small, medium, and large *Sceloporus* species in each bioregion differed from that expected given the frequency of species of each size class in the genus as a whole. Lastly, we used a phylogenetic generalized least square (PGLS) on SVL using a model of Brownian motion. We extracted the residuals of the PGLS and regressed these against the chi‐squared values from the divergent sympatry analysis to correlate body size with our measure of divergent sympatry and taking phylogenetic history into account.

## 3 RESULTS

### 3.1 Patterns of sympatry across bioregions

**Figure 1:**
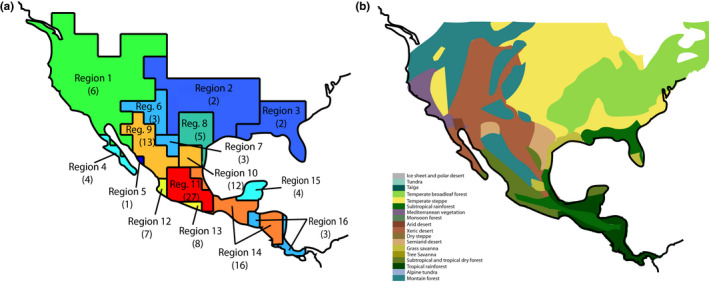
Panel (a) describes the bioregions identified by the Infomap clustering algorithm (Edler et al., 2017). Warmer colors indicate bioregions that possess many Sceloporus species, whereas cooler colors reflect regions with few species. Values in parentheses indicate the number of species found in each region. Note that several species occur in more than one bioregion. Panel (b) represents the different biomes of North America, modified from the original image by Ville Koistinen

**Figure 2:**
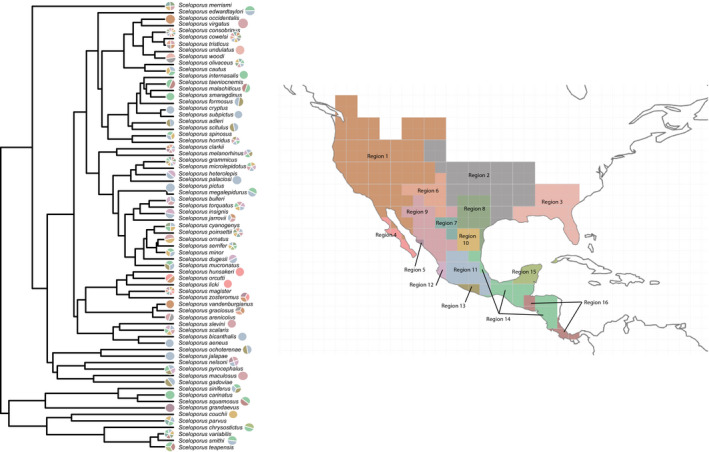
A map showing the recovered bioregions from the clustering analysis. Also shown is the phylogeny used in the analyses modified from Leaché, Banbury (Leaché et al., 2016). Next to the species is a pie chart indicating which bioregion each species belongs to and colors of the pie slices correspond to colors of the bioregions in the map

Using the Infomap Bioregions application and the GBIF occurrence data from 69 species of *Sceloporus*, we identified 16 distinct regions (Figure 1; Table 2). Nearly, all regions contained multiple *Sceloporus* species, with the exception of Region 5 that had a single species (*S. grandaevus*). Conversely, some species (e.g., *S. grammicus* and *S. variabilis*) occurred in more than one bioregion because of their large distributions (Figure 2).

**Table 2:** Biome overlap of each region identified by Infomap Bioregions (Edler et al., 2017) for *Sceloporus* lizards

Bioregion	Number of species	Biome description
Bioregion 1	6 (1S, 2M, 3L)	Montane forest, arid desert, temperate steppe, and Mediterranean vegetation
Bioregion 2	2 (1S, 1M, 0L)	Temperate broadleaf forest and temperate steppe
Bioregion 3	2 (2S, 0M, 0L)	Subtropical rainforest
Bioregion 4	4 (0S, 1M, 3L)	Arid desert and subtropical dry forest
Bioregion 5	1 (1S, 0M, 0L)	Subtropical dry forest
Bioregion 6	3 (0S, 2M, 1L)	Montane forests
Bioregion 7	3 (1S, 1M, 1L)	Arid desert
Bioregion 8	5 (1S, 2M, 2L)	Temperate broadleaf forest, semiarid desert, subtropical dry forest, temperate steppe and arid desert
Bioregion 9	12 (4S, 3M, 5L)	Montane forest, arid desert, and semiarid desert
Bioregion 10	12 (1S, 6M, 5L)	Montane forest, arid desert
Bioregion 11	27 (10S, 10M, 7L)	Subtropical dry forest, montane forest, grass savanna, arid desert, and tropical rainforest
Bioregion 12	7 (0S, 4M, 3L)	Subtropical dry forest
Bioregion 13	8 (2S, 3M, 3L)	Subtropical dry forest
Bioregion 14	16 (3S, 5M, 8L)	Montane forest and tropical rainforest
Bioregion 15	4 (2S, 0M, 2L)	Subtropical dry forest
Bioregion 16	3 (1S, 1M, 1L)	Montane forest and tropical rainforest

Note

In the number of species column, values in parentheses indicate how many species of each size category are found within each region where S = small, *M* = medium, and L = large. See Figure 1 for locations of each Bioregion. See Appendix S1 for more detailed descriptions using the ecoregion classifications of Olson, Dinerstein (Olson et al., 2001).

We found a positive relationship between the number of biomes and number of species across bioregions (Figure 1b; LM: *F*_1,14_ = 11.17, *R* ^2^ = 0.44, *p* = .005). For example, Region 2, with only temperate biomes, and Region 3, with only a single subtropical biome, have only 2 *Sceloporus* species each (Figure 1; *S. consobrinus* and *S. arenicolus* in Region 2; *S. ndulates* and *S. woodi* in Region 3). In contrast, Region 11 which contains a wide range of biomes (e.g., arid deserts, savannas, dry forests, and rainforests) has 27 *Sceloporus* species.

We found a latitudinal gradient where the regions with the fewest species tended to be near the northern part of the genus range, while the regions with the largest numbers of species were located centrally and to the south (Figure 1). For example, Region 11 and Region 14 harbored a large number of species and were closer to the equator than Regions 2 and 3.

**Figure 3:**
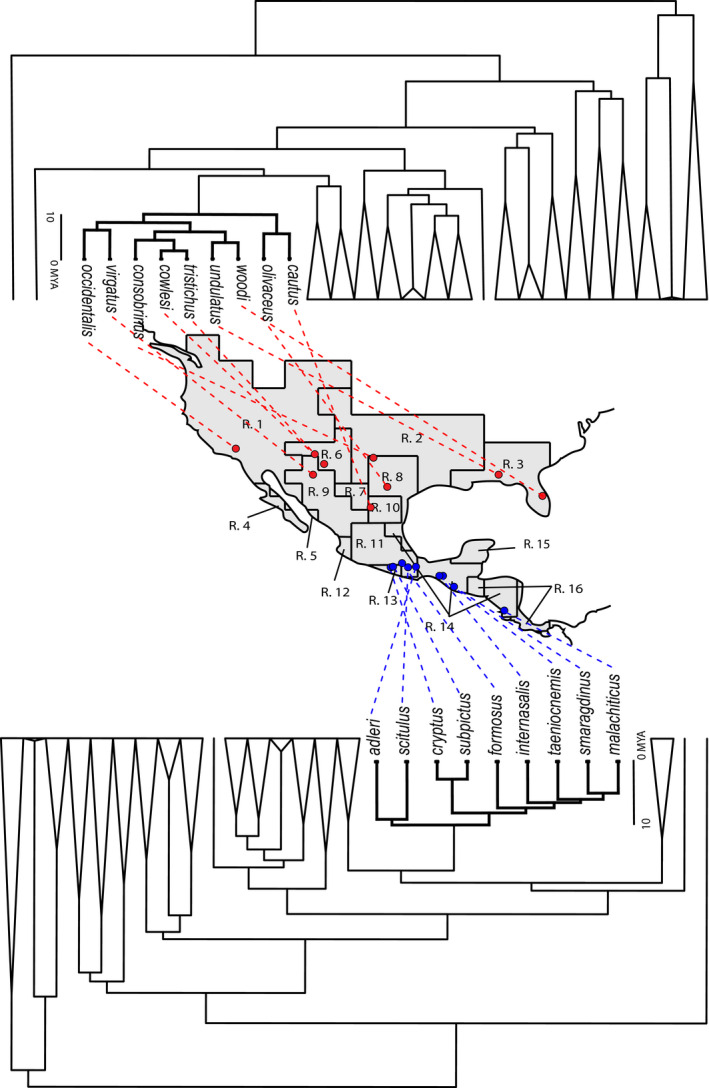
Most closely related Sceloporus species exist in relatively close proximity on a map, as in the lower, blue, formosus clade in this figure. In contrast, species in the undulatus clade (upper clade in red) are widely distributed across the southern United States. Dashed lines link the species name on a phylogeny to the geographic center of their range. Phylogenies were modified from Leaché, Banbury (Leaché et al., 2016). Biogeography of the other Sceloporus clades is presented in Figure S2

**Figure 4:**
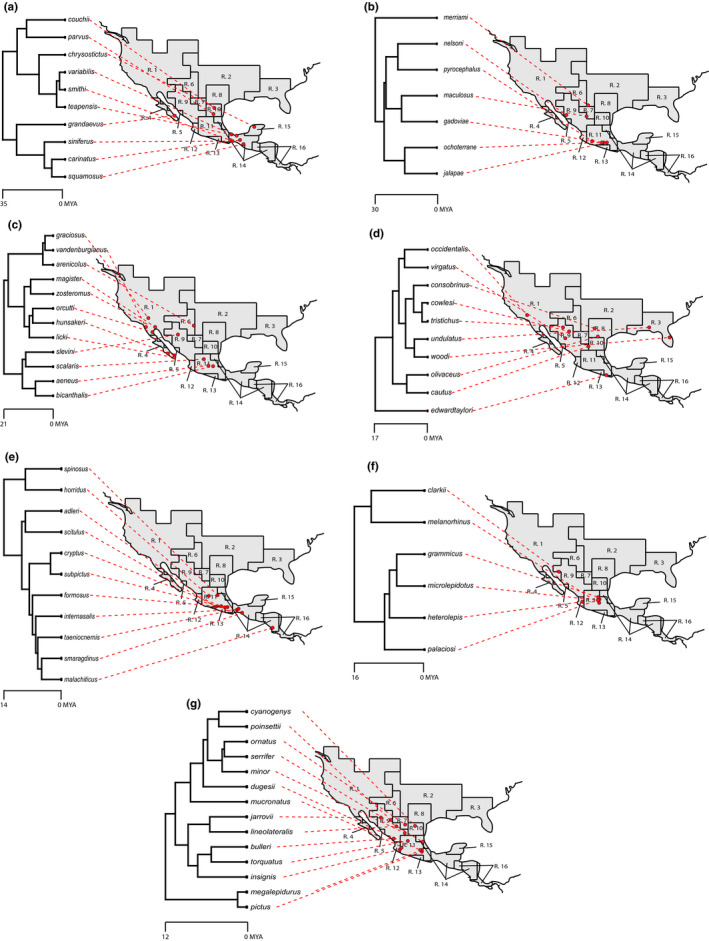
Maps depicting the geographic center for a species’ range separated by clade. Dashed lines are used to link the species with their respective range center. Panel (a) represents the (angustus + siniferus + variabilis) clades, panel (b) represents the (merriami + pyrocephalus + gadoviae + jalapae) clades, panel (c) represents the (graciosus + magister + scalaris) clades, panel (d) represents the (undulatus + S. edwardtaylori) clade, panel (e) represents the (spinosus + formosus) clades, panel (f) represents the (clarkii + grammicus) clades, and panel (g) represents the (megalepidurus + torquatus + poinsettii) clades

Overlaying the Leaché, Banbury (Leaché et al., 2016) phylogeny on the map (Figures 3 and Figure 4), we found that sister species often co‐occurred closely in geographic space. This was also supported by our community structure analysis across bioregions (Table 3). We found that the majority of bioregions show a pattern of phylogenetic clustering indicated by negative standardized effect sizes and small p‐value quantiles. For example, the centers of the ranges for sister species *S. cryptus* and *S. subpictus* are close to each other in geographic space, as are the range centers of sister species *S. adleri* and *S. scitulus* (lower part of Figure 3). Thus, most bioregions contained species from only one or a few clades (Figure 4). We found that only five bioregions showed a moderate pattern of phylogenetic overdispersion, Regions 1, 2, 5, 9, and 15 indicating that these bioregion harbor species that are not closely related. One notable exception was the speciose Region 11 in central Mexico, which contained species from almost every clade (*N* = 27; Figure 4).

**Table 3:** Results from the mean pairwise distance (MPD) community structure analysis. We used the standardized effect size and p‐value quantiles to evaluate the results

	Observed MPD community	Mean MPD in null community	Standardized effect size	*p*‐value quantile
Bioregion 1	53.17	49.27	1.24	0.92
Bioregion 2	56.15	49.03	0.72	0.71
Bioregion 3	11.28	50.97	−3.06	0.01
Bioregion 4	44.81	48.84	−2.02	0.05
bioregion 5	54.56	49.81	0.7	0.75
Bioregion 6	49.12	49.24	−0.02	0.45
Bioregion 7	45.01	49.33	−0.63	0.28
bioregion 8	38.32	48.56	−1.94	0.04
Bioregion 9	59.12	50.62	0.68	0.64
Bioregion 10	27.34	48.34	−2.37	0.01
Bioregion 11	22.16	47.72	−2.47	0.01
Bioregion 12	41.29	48.6	−1.96	0.03
Bioregion 13	47.53	49.62	−0.45	0.27
Bioregion 14	36	48.29	−1.96	0.05
Bioregion 15	60.55	51.18	0.94	0.82
Bioregion 16	35.26	49.05	−1.76	0.05

We found no relationship between the number of species present and land area (Pearson's correlation: ρ = 0.18, *df* = 14, *p* > .05) or latitude (Pearson's correlation: ρ = −0.34, *df* = 14, *p* > .05) of each bioregion. However, the number of species within a bioregion was positively related to our measure of elevation heterogeneity (standard deviation of elevation) (Pearson's correlation: ρ = 0.51, *df* = 14, *p* = .04).

### 3.2 Body size evolution

**Figure 5:**
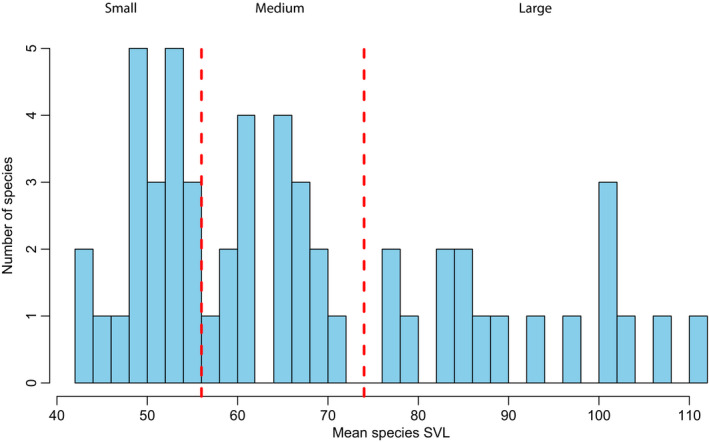
Distribution of body sizes (adult male Snout‐to‐Vent Length) for 69 Sceloporus species. We used the natural breakpoints, vertical red lines, in the histogram to bin species into three size classes

**Figure 6:**
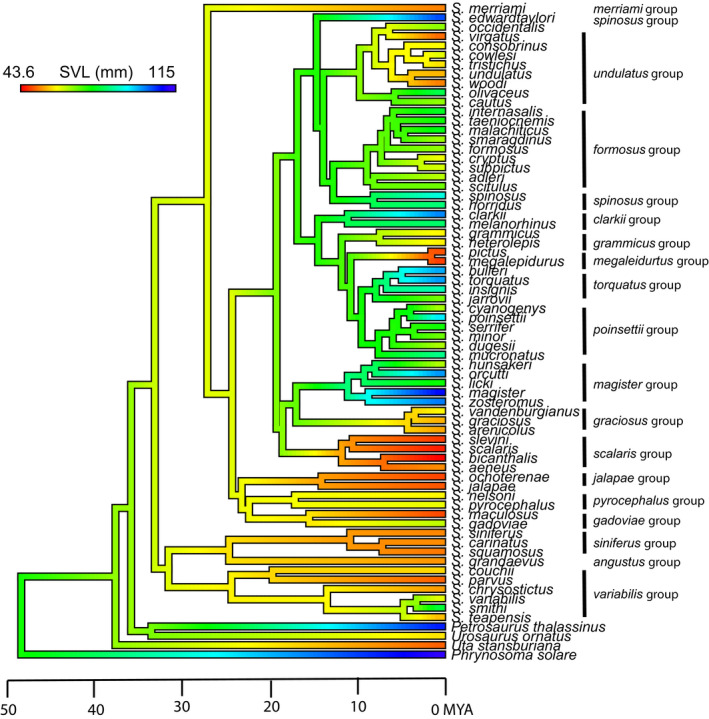
Ancestral reconstruction of Sceloporus and outgroups body size using a Brownian motion model of evolution. Warm colors represent species with small body sizes, while cooler colors represent large‐bodied species. Phylogeny pruned from Leaché, Banbury (Leaché et al., 2016)

We found three discrete size categories for the Sceloporus clade (Figure 5). Additionally, we found that a Brownian motion (BM) model of continuous character evolution (log Likelihood: −445.9, σ^2^ = 8.07) best explained the evolution of body size. The OU model (log Likelihood: −445.9, σ^2^ = 8.07, α = 0.0) was identical to the BM model because the alpha parameter in the OU model is 0 indicating only Brownian motion is driving body size evolution. The Early‐Burst (EB) model did not converge, and therefore, we omitted these results. The ancestor to all *Sceloporus* lizards was likely medium in body size, ~76 mm SVL, with larger bodies evolving only recently in evolutionary time (Figure 6). In addition, body size categories tended to cluster phylogenetically (Figure 6). An estimate of phylogenetic signal confirmed that body size is evolving with high phylogenetic constraint (Pagel's λ = 0.99; Log likelihood = 270.34). Thus, closely related species tend to be similar in body size. Increases in body size occurred abruptly in a few lineages and were then retained over long periods of evolutionary time (Figure 6).

### 3.3 Sympatry and body size evolution

**Figure 7:**
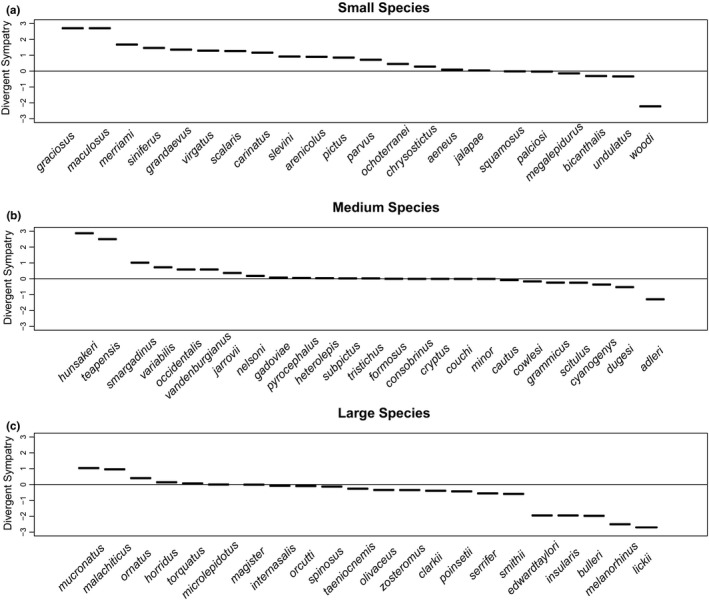
Measures of divergent sympatry for each Sceloporus species. (a) Most small‐bodied Sceloporus species have divergent sympatry, co‐occurring more often with larger congeners. (b) We found no evidence of size assortment by medium‐bodied Sceloporus species. (c) Large‐bodied Sceloporus species show some convergent sympatry, often co‐occurring with similarly sized Sceloporus species

Our chi‐squared analysis found evidence that small *Sceloporus* species are spatially distributed so that they often co‐occur with different sized species (Appendix S2). Conversely, large species were most often found to co‐occur with other large species than by chance alone. More than % of small‐bodied *Sceloporus* species (14 of 22 species) exhibited positive divergent sympatry, occurring more often with medium‐ or large‐bodied congeners than expected given the numbers of species in each size class (Figure 7a). Five of the 22 small‐bodied species exhibited some degree of divergent sympatry, and only 3 species showed convergent sympatry (occurring primarily with other small‐bodied species), and a goodness‐of‐fit test suggested a strong difference between the observed counts (14:5:3) and a uniform expectation (Figure 7a; *χ^2^* = 8.4, *df* = 1, p « 0.01). In contrast, medium‐sized *Sceloporus* species showed only weak evidence of assortment by size, instead occurring with small‐, medium‐, and large‐bodied congeners in proportions roughly comparable to the number of species of each size class available in the genus as a whole (Figure 7b; *χ ^2^* = 0.9, *df* = 1, *p* = .63). More surprisingly, we found that large‐bodied *Sceloporus* species often co‐occur with other large *Sceloporus* species, leading to a significant pattern of negative divergent sympatry (Figure 7c; *χ* ^2^ = 6.2, *df* = 1, *p* = .02). Our correlation analysis between the PGLS residuals of body size and our measure of divergent sympatry confirmed that larger species show a pattern of negative divergent sympatry while smaller species show a pattern of positive divergent sympatry (Pearson's correlation: ρ = −0.41, *df* = 64, *p* = .0008).

At the level of bioregions, we also found evidence of divergent sympatry by small‐bodied *Sceloporus* species and convergent sympatry of large‐bodied *Sceloporus* species. Only Region 3 contained multiple species and was solely comprised of a single size class, while 9 of the 15 regions with multiple species contained all three size categories. We found a disproportionately large number of large‐ and medium‐bodied species and fewer small‐bodied species in Regions 10, 12, and 14. There were no regions with a disproportionately large number of small‐bodied species.

## 4 DISCUSSION

Using public information from large open‐source databases and phylogenetic information, we found several important insights into the geography of congeneric sympatry and phenotypic divergence in *Sceloporus* lizards. First, closely related species tend to be in close geographic proximity (phylogenetic underdispersion) and share similar body sizes, suggesting that they have been evolving gradually. The large number of closely related, sympatric species in ecologically diverse regions, and the positive relationship between habitat heterogeneity and the number of species present in a bioregion suggest that most speciation occurred over short time frames and in near allopatry. One notable exception was the *undulatus* group, which is geographically widespread across the United States despite only minimal speciation. The *undulatus* clade seems to exemplify Rapoport's rule (Stevens, 1989) as the group is found at higher latitudes and species have exceptionally large ranges with only two widespread *undulatus* species occurring in the Southeast United States and two widespread *undulates* species occurring in the Midwest United States. The group seems to have migrated over large distances in a relatively short period of time (Rivera et al., 2020) with few speciation events occurring. Additionally, the group shows little phenotypic diversification (Leaché, 2009) with most species being medium in body size while only two species, *S. woodi* and *S. virgatus*, were small.

Second, we found that the ancestor of all *Sceloporus* was likely a medium‐bodied species with small‐bodied species evolving soon after. The fossil record indicates that the second oldest *Sceloporus* fossil had unicuspid teeth (Holman, 1987) and may have been the ancestor to *S. merriami*, a small species. Furthermore, large body sizes evolved more recently in the clade's history in a few lineages, where it was then retained over long periods of evolutionary time. Putting biogeography and morphological evolution together, we found evidence for high functional diversity and geographic sorting across bioregions. The majority of bioregions had a nearly equal representation of body size categories with the exception of Regions 10 and 12 where medium and large species were overrepresented. Species also seem to sort geographically so that small‐bodied species co‐occur more often with larger‐bodied species, perhaps to reduce ecological competition or interference like in other lizard species (Losos, 1990; Moritz et al., 2018). In contrast, large‐bodied *Sceloporus* species most often co‐occur with other large‐bodied species, perhaps reflecting phylogenetic constraints on body size evolution and limited dispersal. Future studies may want to investigate the relationship between species distributions, body size, and habitat at smaller spatial scales and to explore functional diversity through more detailed analysis of phenotypes.

### 4.1 Bioregions and sympatry

*Sceloporus* species are unevenly distributed across North America, with some geographically large regions, like the southern and western United States, having few species, whereas geographically smaller regions, like central Mexico and south Central America, have many species. This pattern seems to follow the latitudinal diversity gradient where biodiversity increases from poles to the tropics (Hillebrand, 2004) and Rapoport's rule where species at higher latitudes have lower rates of spatial turnover and larger ranges (Stevens, 1989). The *undulatus* clade exemplifies this as these nine northern species span the entire United States. Conversely, species assemblages at lower latitudes tend to have higher richness and turnover rates (Stevens, 1989). This also seems to be the case in *Sceloporus* as the small area from central Mexico to Panama harbors 65 species, over half of the total diversity of the genus. Other factors may also contribute to the high species richness in relatively small bioregions. For example, the Trans‐Mexican volcanic belt (Region 11), which formed during the Eocene and Miocene periods (Ferrari et al., 2012), is a relatively small geographic region that includes several volcanic peaks that rise above 3,000 m, and the region is a biodiversity hotspot with a string of sky islands (Halffter & Morrone, 2017; Mastretta‐Yanes et al., 2015). Likewise, Region 14 harbors 16 species, is close to the equator, and has a large mountain range named Cordillera de Talamanca that traverses Costa Rica and Panama. This high habitat heterogeneity likely contributes to increased allopatry and speciation within these two regions. This was also supported by our correlation analysis showing that regions with more elevation heterogeneity have a higher species richness compared to regions with low elevation heterogeneity. This pattern may also be explained by the elevation diversity gradient where biodiversity is highest at middle elevation (McCain, 2005), but this has yet to be explicitly tested. Additionally, the lower turnover rates in areas close to the equator may because Central American is vastly smaller in area compared to North America so species tend have smaller ranges.

Early research suggested that the genus *Sceloporus* originated in Mexico given the diversity seen today (Hall, 1973). More recent studies, however, found that *Sceloporus* most likely originated much further north in what is now the northern United States and Canada (Lawing et al., 2016). Based on fossil evidence and paleoclimate reconstructions, Mexico was too hot and dry and, therefore, unsuitable for the genus until about 6 million years ago (Lawing et al., 2016). We note that Lawing, Polly (Lawing et al., 2016) estimated the limits of the realized niche using the abiotic BIOCLIM variables and did not include other abiotic or biotic factors that are also driving the evolution of ranges in the lineage. When the global climate cooled later in the Miocene, speciation rates within the genus *Sceloporus* increased (Leaché et al., 2016), which coincides with *Sceloporus* entering central Mexico. Rapid diversification may have been mediated by topographical complexity as land uplift in the volcanic belt began some 20 MYA and continues today (Dimmitt et al., 2015). The complex topography of the Trans‐Mexican volcanic belt would have allowed novel and disparate niches to emerge which *Sceloporus* could have exploited. This would have led to a rapid diversification and build‐up of endemic species assemblages seen today.

In addition to Rapoport's rule, the lack of diversity at higher latitudes may reflect historical events like glacial maxima and shifts in climate regime. If taxa were unable to find refuges from increasing glacier event, species would fail to recolonize and be extirpated (Grubb et al., 1987) leaving only lineages farther south. Second, the emergence of new unsuitable habitat may also contribute to this pattern. *Sceloporus* lizards may have been extirpated in Canada and parts of the United States due to the rise of the Rocky Mountains, which blocked moisture from the midcontinent (Dimmitt et al., 2015). This allowed the Great Plains of North America to emerge but also created more xeric conditions (Dimmitt et al., 2015) that may have excluded *Sceloporus* from this newly formed habitat. The *undulatus* clade may be a secondary rerecolonization of *Sceloporus* to more northern regions that were previously uninhabitable due to shifts in climate regime or glaciers. Speciation in this group would have occurred more gradually as the clade spread across the United States. Whether glacial events, shifts in climate regime, or geological activity contribute to the biogeographic pattern of *Sceloporus* is unknown; however, these would explain the lack of richness in northern North America. Additionally, lizards are generally poor dispersers (Escobedo‐Galván et al., 2011) and take long periods of evolutionary time to occupy new geographic ranges. Thus, large‐bodied *Sceloporus* species may cluster geographically in present time because they are recently evolved and have had less time to disperse. It may also be the case that because larger species have larger home ranges and are therefore more likely to overlap, however this has yet to be studied. Alternatively, large‐bodied lizards may differ in niche breadth compared to their smaller congeners reducing the potential habitats they can use and restricting large‐bodied lizards to particular areas. We have some evidence for this pattern as large‐bodied lizards can be found in montane, subtropical and tropical forests or arid deserts, but this has yet to be studied.

### 4.2 Body size and trait divergence

In general, *Sceloporus* do not show the same pattern of morphological and ecological specialization as other closely related clades (Warheit et al., 1999). By taking our approach, we can begin to identify large‐scale patterns of diversification that are normally difficult to obtain. For example, resource availability may drive small species to be geographically spread to reduce competition. Conversely, large species are often found with other large species and alternative reproductive and foraging strategies may facilitate co‐occurrence. This seems to be the case for two large species, *S. torquatus and S. spinosus*, which are often found in sympatry. Interestingly, their peak mating seasons are separated by several months (Feria‐Ortiz et al., 2001; Valdéz‐González & Ramírez‐Bautista, 2002). *S. spinosus* is oviparous, has peak mating activity in April and May, and eats insects and other invertebrates (Valdéz‐González & Ramírez‐Bautista, 2002). *S. torquatus*, on the other hand, is viviparous, mates in the fall (Feria‐Ortiz et al., 2001) and is omnivorous, increases intake of plant material during the summer, but mainly feeds on insects and other arthropods in early and late season (Búrquez et al., 1986). *Sceloporus* species tend to increase their activity, including foraging, during the peak mating season, and, at least in males, reducing activity during nonbreeding seasons (Rose, 1981). By employing strategies like off‐setting breeding seasons or modification of diet and activity through the year, species may be able to live in sympatry with reduced antagonistic interactions.

A limitation in our study was the lack of intraspecific variation in the body size data. Phenotypic variation has long been known to be an important measure that can affect the response to selective pressures, how species interacts with abiotic and biotic factors, and even community dynamics (Bolnick et al., 2003). Thus, ignoring intraspecific variation may overlook fundamental processes that dictate species assemblages and the ecological breadth of a species (Violle et al., 2012). The lack of intraspecific size data can become an issue for species with large distributions, like *S. grammicus* and *S. variablis*, because populations are bound to differ in body size and sampling effort may not be even across the range. This may have led to incorrect classification of the size, small versus medium, for example, which would affect our chi‐squared analysis, ancestral reconstruction, and understanding of how species’ phenotypes are distributed. This can be overcome by measuring a large number of individuals and equal sampling efforts across the range of the species. In our case, *S. grammicus* and *S. variabilis* both had large sample sizes, 412 and 457 individuals, respectively, but some species, like *S. zosteromus* may be more vulnerable to bias.

### 4.3 A different approach to studying diversification

As large data sets become more accessible through open‐source databases, we can begin to address questions at a larger scale. For example, GBIF is thought to be a good estimate for species richness completeness in both the United States (~75%–80%) and Mexico (~60%) (Meyer et al., 2015) allowing researchers to address questions about all of North America, rather than a small area. Additionally, much of the data that populates large databases come from reputable sources like museums. For example, between 70% and 80% of occurrence data for the clade Reptilia comes from museums vouchers and are accurate in terms of species identification and locality.

Although little work has been done describing bioregions in terms of ectotherms in North America, a study by Burbrink and Gehara (Burbrink & Gehara, 2018) found that the clade *Lampropeltis*, New World kingsnakes, showed a similar composition of bioregions as found in our study. For example, we found distinct bioregions in the west coast, east coast, central, and southwest United States and this same pattern was found by Burbrink and Gehara (Burbrink & Gehara, 2018); however, the size of the bioregions slightly differed. Bioregions differed dramatically in Mexico and across Central America. We found ~ 10 bioregions across southern North America and Central America, while Burbrink and Gehara (Burbrink & Gehara, 2018) only found two. This difference in the number of bioregions is likely explained by the disparity in diversity between *Sceloporus* and *Lampropeltis*. There are ~ 25 species of *Lampropeltis* across the Americas, while *Sceloporus* has ~ 100 species with a majority of the diversity centered in Mexico. The great diversity of *Sceloporus* coupled with high endemism may also explain the fine partitioning of bioregions found in Mexico compared to the two large regions found by Burbrink and Gehara (Burbrink & Gehara, 2018).

Many insular examples exist of closely related species occupying a single geographic region and exhibiting the phenotypic consequences that arise from such sympatry (Lack, 1947; Losos et al., 1998; Seehausen, 2006). These examples have led to groundbreaking work in evolutionary biology and pushed the field forward, yet they may represent the exception rather than the rule. Here, we emphasize and promote the use of open‐source databases to perform macroscale analyses across large geographic areas that were previously difficult to achieve. Additionally, large‐scale analyses provide a novel view into continental systems to gain new insights on macroevolutionary processes that create large‐scale patterns of diversity.

## CONFLICT OF INTEREST

None declared.

## AUTHOR CONTRIBUTIONS

**Julio Rivera:** Formal analysis (equal); Writing‐original draft (equal); Writing‐review & editing (lead). **Heather N. Rich:** Conceptualization (lead); Formal analysis (equal); Writing‐original draft (lead). **Michelle Lawling:** Conceptualization (equal); Formal analysis (supporting); Writing‐review & editing (supporting). **Michael S. Rosenberg:** Conceptualization (supporting); Formal analysis (supporting); Writing‐review & editing (supporting). **Emilia P Martins:** Conceptualization (lead); Writing‐review & editing (equal).

## Supporting information

Appendix S1

## Data Availability

All data in this study were downloaded from open‐source material online. See *Methods* section of the manuscript for details. Generally, occurrences were downloaded from GBIF, and size data were collected from published and unpublished literature.
