# Supplementary material for: Occurrence data uncover patterns of allopatric divergence and interspecies interactions in the evolutionary history of Sceloporus lizards
Source: Ecol Evol. 2021 Feb 8;11(6):2796–813. doi: 10.1002/ece3.7237 (PMC7981219; doi:10.1002/ece3.7237)
Supplement: Supplementary file 1 — Appendix S1 [file ECE3-11-2796-s001.docx]

Appendix S1. A description of the ecoregion composition within each bioregion. Ecoregions were derived from Olson, Dinerstein (57)

| Bioregion | Ecoregion composition |
| --- | --- |
| Bioregion 1 | North Cascades, Coast Range, Willamette Valley, Cascades, Eastern Cascades Slopes and Foothills, Klamath Mountains, Southern and Central California Chaparral and Oak Woodlands, Sierra Nevada, Central California Valley, Mojave Basin and Range, Sonoran Basin and Range, Arizona/New Mexico Mountains, Wasatch and Uinta Mountains, Colorado Plateaus, Southern Rockies, Columbia Plateau, Blue Mountains, Northern Basin and Range, Idaho Batholith, Snake River Plain, Middle Rockies, Northwestern Glaciated Plains, and Northwestern Great Plains. |
| Bioregion 2 | Lake Agassiz Plain, Northern Glaciated Plains, Nebraska Sand Hills, Western Corn Belt Plains, Middle Rockies, Western High Plains, Central Great Plains, Flint Hills, Central Irregular Plains, Ozark Highlands, Central Corn Belt Plains, Interior River Valleys and Hills, Mississippi Valley Loess Plains, Mississippi Alluvial Plain, Flint Hills, Central Great Plains, and Western High Plains. |
| Bioregion 3 | Middle Atlantic Coastal Plain, Southeastern Plains, Piedmont, Blue Ridge, Ridge and Valley, Southwestern Appalachians, Southeastern Plains, and Interior Low Plateaus, Southern Coastal Plain, and Southern Florida Coastal Plain. |
| Bioregion 4 | Baja Californian Desert, California Coastal Sage, Chaparral, and Oak Woodlands, Sonoran Desert, Los Cabos Plains and Hills, and La Laguna Mountains. |
| Bioregion 5 | Sinaloa Coastal Plain, Sinaloa and Sonora Hills and Canyons. |
| Bioregion 6 | Arizona/New Mexico Plateau, Arizona/New Mexico Mountains, Chihuahuan Deserts, Southern Rockies, and Western High Plains. |
| Bioregion 7 | Southern Texas Plains, Edwards Plateau, and Chihuahuan Deserts. |
| Bioregion 8 | Western Gulf Coastal Plain, South Central Plains, East Central Texas Plains,  Central Oklahoma/Texas Plains, Edwards Plateau, Central Great Plains, and Southern Texas Plains. |
| Bioregion 9 | Arizona/New Mexico Mountains, Arizona/New Mexico Plateau, Chihuahuan Deserts, Piedmonts and Plains, Sierra Madre Occidental, Sinaloa and Sonora Hills and Canyons, Sinaloa Coastal Plain, Sonoran Desert, and Southern Texas Plains/Interior Plains and Hills. |
| Bioregion 10 | Chihuahuan Desert, Southern Texas Plains/Interior Plains and Hills, Sierra Madre Oriental, and Western Gulf Coastal Plain. |
| Bioregion 11 | Jalisco/Nayarit Hills and Plains, South Pacific Hills and Piedmonts, Sierras of Jalisco and Michoacán, Hills and Sierras, Hills and Interior Plains, Sierra Madre Occidental, Sierra Madre Oriental, Sierras of Jalisco and Michoacán, Interior Plains and Piedmonts, Sinaloa and Sonora Hills and Canyons, and Chihuahuan Desert. |
| Bioregion 12 | Hills and Interior Plains, Piedmonts and Plains, Chihuahuan Desert, and Sierra Madre Oriental. |
| Bioregion 13 | Balsas Depression, Sierras of Guerrero and Oaxaca, and South Pacific Hills and Piedmonts. |
| Bioregion 14 | Valleys and Depressions, Sierras of Guerrero and Oaxaca, Hills with Medium and High Evergreen Tropical Forest, Los Tuxtlas Sierra, Gulf of Mexico Coastal Plain, Chiapas Highlands, Chiapas Depression, Central American Sierra Madre, Central American dry forests, Central American pine-oak forests, Gulf of Fonseca mangroves, Northern Dry Pacific Coast mangroves, Central American Atlantic moist forests, Central American montane forests, Miskito pine forests, Mosquitia-Nicaraguan Caribbean Coast mangroves, and Northern Honduras mangroves. |
| Bioregion 15 | Northwestern Yucatan Plain, Plain with Low and Medium Deciduous Tropical Forest, Plain with Medium and High Semi-Evergreen Tropical Forest, and Hills with High and Medium Semi-Evergreen Tropical Forest. |
| Bioregion 16 | Chocó-Darién moist forests, Eastern Panamanian montane forests, Isthmian-Atlantic moist forests, Isthmian-Pacific moist forests, Talamancan montane forests, Panamanian dry forests, Bocas del Toro-San Bastimentos Island-San Blas mangroves, Gulf of Panama mangroves, Moist Pacific Coast mangroves, Cocos Island moist forests, Costa Rican seasonal moist forests, Central American dry forests, Rio Negro-Rio San Sun mangroves, and Southern Dry Pacific Coast mangroves. |

Appendix S2. A list of focal species, their body size category, and species that co-occur within 5 km of the focal species.

| Focal species | Size | Species in sympatry |
| --- | --- | --- |
| *adleri* | medium | *formosus, grammicus, horridus, mucronatus, scitulus, siniferus* |
| *aeneus* | small | *ochoterenae, scalaris, pyrocephalus, bicanthalis, dugesii, heterolepis, grammicus, gadoviae, parvus, horridus, mucronatus, spinosus, microlepidotus, torquatus, melanorhinus, variabilis, palaciosi* |
| *arenicolus* | small | *consobrinus, cowlesi* |
| *bicanthalis* | small | *formosus, scalaris, serrifer, cryptus, aeneus, grammicus, siniferus, parvus, horridus, torquatus, spinosus, microlepidotus, mucronatus, megalepidurus, variabilis, palaciosi* |
| *bulleri* | large | *clarkii, scalaris, dugesii, insignis, heterolepis, grammicus, horridus, torquatus, jarrovii, microlepidotus, nelsoni, melanorhinus, poinsettii* |
| *carinatus* | small | *taeniocnemis, serrifer, squamosus, siniferus, smaragdinus, teapensis, melanorhinus, smithi, variabilis, internasalis* |
| *cautus* | medium | *cowlesi, scalaris, serrifer, consobrinus, minor, grammicus, ornatus, parvus, torquatus, spinosus, microlepidotus, olivaceus, cyanogenys, variabilis, poinsettii* |
| *chrysostictus* | small | *serrifer, teapensis, variabilis* |
| *clarkii* | large | *cowlesi, bulleri, magister, scalaris, consobrinus, tristichus, horridus, slevini, spinosus, jarrovii, nelsoni, melanorhinus, poinsettii, virgatus* |
| *consobrinus* | medium | *nelsoni, cautus, cowlesi, magister, serrifer, couchii, undulatus, merriami, torquatus, microlepidotus, olivaceus, poinsettii, cyanogenys, variabilis, clarkii, minor, ornatus, grammicus, tristichus, graciosus, arenicolus, parvus, horridus, slevini, jarrovii, maculosus, virgatus* |
| *couchii* | medium | *cowlesi, scalaris, serrifer, consobrinus, minor, grammicus, ornatus, parvus, merriami, spinosus, microlepidotus, torquatus, olivaceus, cyanogenys, variabilis, poinsettii* |
| *cowlesi* | medium | *clarkii, magister, couchii, consobrinus, ornatus, grammicus, poinsettii, tristichus, parvus, slevini, merriami, jarrovii, olivaceus, arenicolus, cyanogenys, virgatus, cautus* |
| *cryptus* | medium | *formosus, bicanthalis, grammicus, horridus, microlepidotus, mucronatus, variabilis, siniferus* |
| *cyanogenys* | medium | *cowlesi, scalaris, serrifer, couchii, consobrinus, minor, grammicus, ornatus, parvus, torquatus, spinosus, microlepidotus, olivaceus, poinsettii, variabilis, cautus* |
| *dugesii* | medium | *scalaris, bulleri, pyrocephalus, aeneus, heterolepis, grammicus, gadoviae, horridus, torquatus, spinosus, microlepidotus, nelsoni* |
| *edwardtaylori* | large | *grammicus, spinosus, microlepidotus, melanorhinus, smithi, variabilis, siniferus* |
| *formosus* | medium | *jalapae, melanorhinus, serrifer, gadoviae, torquatus, spinosus, microlepidotus, scitulus, variabilis, ochoterenae, bicanthalis, subpictus, grammicus, adleri, scalaris, cryptus, horridus, pictus, mucronatus, megalepidurus, siniferus* |
| *gadoviae* | medium | *ochoterenae, formosus, pyrocephalus, dugesii, aeneus, grammicus, jalapae, horridus, mucronatus, spinosus, microlepidotus, pictus, torquatus, melanorhinus, megalepidurus, variabilis, siniferus* |
| *graciosus* | small | *magister, consobrinus, vanderburgianus, tristichus, occidentalis, orcutti* |
| *grammicus* | medium | *jalapae, melanorhinus, smithi, cautus, cowlesi, bulleri, magister, couchii, serrifer, dugesii, aeneus, heterolepis, gadoviae, merriami, spinosus, microlepidotus, torquatus, olivaceus, cyanogenys, poinsettii, scitulus, palaciosi, ochoterenae, formosus, variabilis, bicanthalis, edwardtaylori, insignis, consobrinus, minor, ornatus, subpictus, adleri, scalaris, cryptus, pyrocephalus, parvus, horridus, jarrovii, pictus, maculosus, mucronatus, megalepidurus, siniferus* |
| *grandaevus* | small | *zosteromus, hunsakeri, orcutti* |
| *heterolepis* | medium | *scalaris, bulleri, dugesii, aeneus, grammicus, horridus, torquatus, microlepidotus* |
| *horridus* | large | *jalapae, nelsoni, melanorhinus, bulleri, magister, dugesii, heterolepis, aeneus, gadoviae, torquatus, spinosus, microlepidotus, poinsettii, scitulus, variabilis, palaciosi, clarkii, formosus, ochoterenae, bicanthalis, insignis, consobrinus, grammicus, adleri, scalaris, cryptus, pyrocephalus, jarrovii, pictus, mucronatus, megalepidurus, virgatus, siniferus* |
| *hunsakeri* | medium | *licki, magister, grandaevus, zosteromus, orcutti* |
| *insignis* | large | *scalaris, bulleri, grammicus, horridus, torquatus, microlepidotus* |
| *internasalis* | large | *taeniocnemis, serrifer, carinatus, squamosus, smaragdinus, teapensis, melanorhinus, variabilis, siniferus* |
| *jalapae* | small | *formosus, scalaris, serrifer, subpictus, grammicus, gadoviae, horridus, spinosus, microlepidotus, pictus, mucronatus, megalepidurus, variabilis, siniferus* |
| *jarrovii* | medium | *clarkii, cowlesi, bulleri, magister, scalaris, consobrinus, ornatus, grammicus, nelsoni, horridus, slevini, torquatus, microlepidotus, maculosus, poinsettii, virgatus* |
| *licki* | large | *magister, zosteromus, hunsakeri, orcutti* |
| *maculosus* | small | *magister, consobrinus, ornatus, grammicus, jarrovii, poinsettii* |
| *magister* | large | *occidentalis, nelsoni, cowlesi, merriami, olivaceus, zosteromus, poinsettii, clarkii, licki, consobrinus, grammicus, tristichus, graciosus, vanderburgianus, parvus, horridus, slevini, jarrovii, maculosus, hunsakeri, orcutti* |
| *malachiticus* | large | *squamosus, variabilis* |
| *megalepidurus* | small | *formosus, scalaris, serrifer, bicanthalis, subpictus, grammicus, jalapae, gadoviae, parvus, horridus, torquatus, spinosus, microlepidotus, pictus, mucronatus, variabilis, siniferus* |
| *melanorhinus* | large | *teapensis, nelsoni, smithi, serrifer, bulleri, aeneus, gadoviae, torquatus, spinosus, microlepidotus, scitulus, variabilis, palaciosi, clarkii, formosus, ochoterenae, carinatus, edwardtaylori, grammicus, siniferus, taeniocnemis, scalaris, pyrocephalus, horridus, mucronatus, internasalis* |
| *merriami* | small | *cowlesi, magister, couchii, consobrinus, ornatus, grammicus, parvus, olivaceus, poinsettii* |
| *microlepidotus* | large | *jalapae, melanorhinus, smithi, cautus, serrifer, bulleri, couchii, dugesii, aeneus, heterolepis, gadoviae, torquatus, spinosus, olivaceus, cyanogenys, poinsettii, variabilis, palaciosi, ochoterenae, formosus, bicanthalis, insignis, edwardtaylori, consobrinus, minor, grammicus, scalaris, cryptus, pyrocephalus, parvus, horridus, jarrovii, pictus, mucronatus, megalepidurus, siniferus* |
| *minor* | medium | *scalaris, serrifer, couchii, consobrinus, ornatus, grammicus, poinsettii, parvus, torquatus, spinosus, microlepidotus, olivaceus, mucronatus, cyanogenys, variabilis, cautus* |
| *mucronatus* | large | *jalapae, melanorhinus, serrifer, aeneus, gadoviae, torquatus, spinosus, microlepidotus, scitulus, variabilis, palaciosi, ochoterenae, formosus, bicanthalis, minor, subpictus, grammicus, adleri, scalaris, cryptus, pyrocephalus, parvus, horridus, pictus, megalepidurus, siniferus* |
| *nelsoni* | medium | *clarkii, scalaris, magister, bulleri, dugesii, consobrinus, horridus, torquatus, spinosus, jarrovii, melanorhinus, virgatus* |
| *occidentalis* | medium | *magister, vanderburgianus, zosteromus, graciosus, orcutti* |
| *ochoterenae* | small | *formosus, scalaris, pyrocephalus, aeneus, grammicus, siniferus, gadoviae, variabilis, horridus, mucronatus, spinosus, microlepidotus, torquatus, melanorhinus, scitulus, palaciosi* |
| *olivaceus* | large | *cowlesi, magister, scalaris, couchii, serrifer, consobrinus, minor, grammicus, ornatus, poinsettii, parvus, merriami, spinosus, microlepidotus, torquatus, cyanogenys, variabilis, cautus* |
| *orcutti* | large | *licki, magister, vanderburgianus, grandaevus, occidentalis, zosteromus, graciosus, hunsakeri* |
| *ornatus* | large | *cowlesi, scalaris, serrifer, couchii, consobrinus, minor, grammicus, poinsettii, parvus, merriami, spinosus, jarrovii, olivaceus, maculosus, cyanogenys, cautus* |
| *palaciosi* | small | *ochoterenae, scalaris, bicanthalis, aeneus, grammicus, horridus, torquatus, spinosus, microlepidotus, mucronatus, melanorhinus, variabilis* |
| *parvus* | small | *cautus, cowlesi, magister, serrifer, couchii, aeneus, merriami, spinosus, microlepidotus, torquatus, olivaceus, poinsettii, cyanogenys, variabilis, bicanthalis, minor, ornatus, grammicus, consobrinus, scalaris, mucronatus, megalepidurus* |
| *pictus* | small | *formosus, grammicus, jalapae, gadoviae, horridus, torquatus, spinosus, microlepidotus, mucronatus, megalepidurus, variabilis* |
| *poinsettii* | large | *cautus, cowlesi, magister, bulleri, couchii, serrifer, merriami, spinosus, microlepidotus, torquatus, olivaceus, cyanogenys, variabilis, clarkii, consobrinus, ornatus, grammicus, minor, tristichus, scalaris, parvus, horridus, slevini, jarrovii, maculosus, virgatus* |
| *pyrocephalus* | medium | *ochoterenae, dugesii, aeneus, grammicus, gadoviae, horridus, mucronatus, torquatus, microlepidotus, melanorhinus, siniferus* |
| *scalaris* | small | *jalapae, nelsoni, melanorhinus, cautus, serrifer, bulleri, couchii, dugesii, heterolepis, aeneus, torquatus, spinosus, microlepidotus, olivaceus, poinsettii, cyanogenys, variabilis, palaciosi, clarkii, formosus, ochoterenae, bicanthalis, insignis, minor, ornatus, grammicus, parvus, horridus, jarrovii, mucronatus, megalepidurus* |
| *scitulus* | medium | *ochoterenae, formosus, grammicus, horridus, adleri, mucronatus, melanorhinus, siniferus* |
| *serrifer* | large | *jalapae, teapensis, melanorhinus, cautus, couchii, torquatus, spinosus, microlepidotus, olivaceus, poinsettii, cyanogenys, variabilis, formosus, carinatus, bicanthalis, minor, consobrinus, grammicus, ornatus, siniferus, chrysostictus, taeniocnemis, scalaris, parvus, mucronatus, megalepidurus, internasalis* |
| *siniferus* | small | *jalapae, teapensis, melanorhinus, smithi, serrifer, gadoviae, spinosus, microlepidotus, scitulus, variabilis, ochoterenae, formosus, carinatus, edwardtaylori, bicanthalis, grammicus, adleri, smaragdinus, taeniocnemis, pyrocephalus, cryptus, squamosus, horridus, mucronatus, megalepidurus, internasalis* |
| *slevini* | small | *clarkii, cowlesi, magister, consobrinus, jarrovii, poinsettii, virgatus* |
| *smaragdinus* | medium | *taeniocnemis, carinatus, squamosus, internasalis, variabilis, siniferus* |
| *smithi* | large | *carinatus, edwardtaylori, grammicus, spinosus, microlepidotus, melanorhinus, variabilis, siniferus* |
| *spinosus* | large | *jalapae, nelsoni, melanorhinus, smithi, cautus, serrifer, couchii, dugesii, aeneus, gadoviae, torquatus, microlepidotus, olivaceus, poinsettii, cyanogenys, variabilis, palaciosi, ochoterenae, formosus, clarkii, bicanthalis, edwardtaylori, minor, ornatus, grammicus, subpictus, scalaris, parvus, horridus, pictus, mucronatus, megalepidurus, siniferus* |
| *squamosus* | small | *carinatus, siniferus, smaragdinus, malachiticus, variabilis, internasalis* |
| *subpictus* | medium | *formosus, grammicus, jalapae, spinosus, mucronatus, megalepidurus* |
| *taeniocnemis* | large | *serrifer, carinatus, internasalis, smaragdinus, teapensis, melanorhinus, variabilis, siniferus* |
| *teapensis* | medium | *taeniocnemis, serrifer, carinatus, siniferus, chrysostictus, melanorhinus, variabilis, internasalis* |
| *torquatus* | large | *nelsoni, melanorhinus, cautus, serrifer, bulleri, couchii, dugesii, aeneus, heterolepis, gadoviae, spinosus, microlepidotus, olivaceus, cyanogenys, poinsettii, variabilis, palaciosi, ochoterenae, formosus, bicanthalis, insignis, minor, consobrinus, grammicus, scalaris, pyrocephalus, parvus, horridus, jarrovii, pictus, mucronatus, megalepidurus* |
| *tristichus* | medium | *clarkii, cowlesi, magister, consobrinus, graciosus, poinsettii* |
| *undulatus* | small | *consobrinus, woodi* |
| *vanderburgianus* | medium | *magister, occidentalis, zosteromus, graciosus, orcutti* |
| *variabilis* | medium | *jalapae, teapensis, melanorhinus, smithi, cautus, serrifer, couchii, aeneus, gadoviae, torquatus, spinosus, microlepidotus, olivaceus, cyanogenys, poinsettii, palaciosi, ochoterenae, formosus, carinatus, bicanthalis, edwardtaylori, consobrinus, minor, grammicus, siniferus, smaragdinus, chrysostictus, taeniocnemis, scalaris, cryptus, squamosus, parvus, horridus, pictus, malachiticus, mucronatus, megalepidurus, internasalis* |
| *virgatus* | small | *clarkii, cowlesi, consobrinus, slevini, horridus, jarrovii, nelsoni, poinsettii* |
| *woodi* | small | *undulatus* |
| *zosteromus* | large | *licki, magister, vanderburgianus, grandaevus, occidentalis, hunsakeri, orcutti* |
